# Supplementary material for: Avoiding dynastic, assortative mating, and population stratification biases in Mendelian randomization through within-family analyses
Source: Nat Commun. 2020 Jul 14;11:3519. doi: 10.1038/s41467-020-17117-4 (PMC7360778; doi:10.1038/s41467-020-17117-4)
Supplement: Supplementary file 1 — Supplementary information [file 41467_2020_17117_MOESM1_ESM.docx]

**Supplementary Information**

**Brumpton et al.**

**Supplementary Note 1: Possible confounders of genetic associations**

**Assortative mating**: when individuals choose their partners non-randomly, so are more alike or selected on a particular trait than would be expected. This can occur because people select on a specific trait, e.g. if tall women prefer to marry tall men, people who drink alcohol choose partners who also drink,^8^ or because of social homogamy where people select partners who have a similar environmental background to themselves, e.g. if educated women select men with a similar amount of education as themselves who happen to be taller.^71^

**Dynastic effects (genetic nurture)**: when parental genotype affects offspring outcomes through pathways other than via offspring genotype. For example, if more educated parents support their offspring’s education, or if parents smoking positively or negatively affected the likelihood of their offspring smoking. An example of dynastic effects are passive gene-environmental correlations.^11,16,17^ Other relationships, such as grandparents, uncles/aunts and cousins may affect the offspring’s phenotype – these can also be thought of as a form of dynastic effect.

**Fine scale population structure**: when subtle difference in ancestry are associated with offspring phenotypes. For example, on average individuals from the north and west of England are poorer and are less educated, there are also geographic gradients in the distribution of education associated variants.^13^

**Supplementary Note 2: Equivalence of the first difference and fixed effects estimators when estimating within-family Mendelian randomization models.**

The general model as describe above is:

|  | $x_{k,i}=\gamma_{0}+\gamma_{1,j}g_{k,i,j}+f_{k}+u_{k,i}$ | (10) |
| --- | --- | --- |

and

|  | $y_{k,i}=\beta_{0}+\beta_{1}g_{k,i,j}+f_{k}+v_{k,i}$. | (11) |
| --- | --- | --- |

$f_{k}$ is a family-level confounder, $f_{k}=f\left( G_{k}, NG_{k} \right)$ where $G_{k}$ is a family-level genetic component and $NG_{k}$ is a family-level non-genetic component, and $g_{k,i,j}=f^{*}\left( G_{k} \right)$. This data generating process invalidates the Mendelian randomization assumptions when using data from unrelated individuals because of the correlation between $g_{k,i,j}$and $f_{k}$.

With two siblings the model can be written as:

|  | $x_{k,1}=\gamma_{0}+\gamma_{1,j}g_{k,1,j}+f_{k}+u_{k,1}$ | (12) |
| --- | --- | --- |
|  | $x_{k,2}=\gamma_{0}+\gamma_{1,j}g_{k,2,j}+f_{k}+u_{k,2}$ | (13) |
|  | $y_{k,1}=\beta_{0}+\beta_{1}x_{k,1,j}+f_{k}+v_{k,1}$ | (14) |
|  | $y_{k,2}=\beta_{0}+\beta_{1}x_{k,2,j}+f_{k}+v_{k,2}$. | (15) |

*Fixed effects estimation*

Fixed effects estimation estimates the model:

|  | $x_{k,i}=\gamma_{0}+\gamma_{1,j}g_{k,i,j}+cI_{k}+u_{k,i,j}$ | (16) |
| --- | --- | --- |

and

|  | $y_{k,i}=\beta_{0}+\beta_{1}x_{k,i,j}+cI_{k}+v_{k,i,j}$. | (17) |
| --- | --- | --- |

Where $I_{F}$ is a set of family-level indicator variables that takes one value for each family (i.e. a set of extra indicator variables, the same size as the number of families in the model, each one of which takes 1 for one family and 0 for all other families). This model is estimated by taking the deviation from the family-level mean (denoted by $\bar{var_{k}}$) for each observation. Under this method of estimation our model becomes:

|  | $x_{k,i}-\bar{x_{k}}=\gamma_{1,j}g_{k,i,j}-\gamma_{1,j}\bar{g_{k,j}}+f_{k}-\bar{f_{k}}+u_{k,i,j}-\bar{u_{k,j}}$  $=\gamma_{1,j}\left( g_{k,i,j}-\bar{g_{k,j}} \right)+\left( u_{k,i,j}-\bar{u_{k,j}} \right)$ | (18) |
| --- | --- | --- |

and

|  | $y_{k,i}-\bar{y_{k}}=\Gamma_{1}g_{k,i,j}-\Gamma_{1,j}\bar{g_{k,j}}+f_{k}-\bar{f_{k}}+v_{k,i,j}-\bar{v_{k,j}}$.  $=\Gamma_{1}\left( g_{k,i,j}-\bar{g_{k,j}} \right)+\left( v_{k,i,j}-\bar{v_{k,j}} \right)$ | (19) |
| --- | --- | --- |

This estimator can now be consistently estimated using Mendelian randomization as $\left( g_{k,i,j}-\bar{g_{k,j}} \right)$ is independent of the family effect. This can be done by estimating:

|  | $x_{k,i}-\bar{x_{k}}=\gamma_{1,j}g_{k,i,j}-\gamma_{1,j}\bar{g_{k,j}}+u_{k,i,j}-\bar{u_{k,j}}$  $x_{k,i}^{*}=\gamma_{1,j}g_{k,i,j}^{*}+u_{k,i,j}^{*}$ | (20) |
| --- | --- | --- |

and

|  | $y_{k,i}-\bar{y_{k}}=\Gamma_{j}g_{k,i,j}-\Gamma_{1,j}\bar{g_{k,j}}+v_{k,i,j}-\bar{v_{k,j}}$  $y_{k,i}^{*}=\Gamma_{j}g_{k,i,j}^{*}+v_{k,i,j}^{*}$ | (21) |
| --- | --- | --- |

The two-sample MR estimator is then obtained from;

$$\hat{\Gamma}_{j}= \beta_{1}\hat{\gamma}_{j}+\omega_{j}$$

*First difference (FD) estimation*

A special case of the fixed effect estimator is the first difference estimator with two siblings. When the first difference is taken the model becomes:

|  | $x_{k,1}-x_{k,2}=\gamma_{1,j}g_{k,1,j}-\gamma_{1,j}g_{k,2,j}+f_{k}-f_{k}+u_{k,1,j}-u_{k,2,j}$  $=\gamma_{1,j}\left( g_{k,1,j}-g_{k,2,j} \right)+\left( u_{k,1,j}-u_{k,2,j} \right)$ | (22) |
| --- | --- | --- |

and

|  | $y_{k,1}-y_{k,2}=\Gamma_{1,j}g_{k,1,j}-\Gamma_{1,j}g_{k,2,j}+f_{k}-f_{k}+v_{k,1,j}-v_{k,2,j}$  $=\Gamma_{1,j}\left( g_{k,1,j}-g_{k,2,j} \right)+\left( v_{k,1,j}-v_{k,2,j} \right)$. | (23) |
| --- | --- | --- |

As $(g_{k,1}-g_{k,2})$ is uncorrelated with $\left( u_{k,1,j}-u_{k,2,j} \right)$ this model can now be consistently estimated in a two-sample MR estimation by:

|  | $x_{k,1}-x_{k,2}=\gamma_{1,j}\left( g_{k,1,j}-g_{k,2,j} \right)+\left( u_{k,1,j}-u_{k,2,j} \right)$  $x_{k}^{'}=\gamma G_{k,j}^{'}+u_{x,k,j}^{'}$ | (24) |
| --- | --- | --- |

and

|  | $y_{k,1}-y_{k,2}=\Gamma_{1,j}\left( g_{k,1,j}-g_{k,2,j} \right)+\left( v_{k,1,j}-v_{k,2,j} \right)$  $y_{k}^{'}=\Gamma g_{k,j}^{'}+v_{y,k,j}^{'}$. | (25) |
| --- | --- | --- |

The two-sample MR estimation of $\hat{\beta}_{1}$ can then be calculated in the same way as above.

*Equivalence of the first difference and fixed effects estimators*

When there are exactly two individuals in each family these two methods of estimation will give the same result.

For $i=1$ the variables in the fixed effects estimator are

|  | $x_{k,1}^{*}= x_{k,1}-\bar{x_{k}}= x_{k,1}-\frac{1}{2}\left( x_{k,1}+x_{k,2} \right)= \frac{1}{2}\left( x_{k,1}-x_{k,2} \right)$ | (26) |
| --- | --- | --- |

|  | $y_{k,1}^{*}= y_{k,1}-\bar{y_{k}}= y_{k,1}-\frac{1}{2}\left( y_{k,1}+y_{k,2} \right)= \frac{1}{2}\left( y_{k,1}-y_{k,2} \right)$ | (27) |
| --- | --- | --- |

|  | $g_{k,1,j}^{*}= g_{k,1,j}-\bar{g_{k,j}}= g_{k,1,j}-\frac{1}{2}\left( g_{k,1,j}+g_{k,2,j} \right)= \frac{1}{2}\left( g_{k,1,j}-g_{k,2,j} \right)$ | (28) |
| --- | --- | --- |

Therefore, the fixed effects estimator can be written as:

|  | $\frac{1}{2}\left( x_{k,1}-x_{k,2} \right)=\gamma_{1,j}\frac{1}{2}\left( g_{k,1,j}+g_{k,2,j} \right)+\frac{1}{2}\left( u_{k,1,j}-u_{k,2,j} \right)$  ${\frac{1}{2}x}_{k}^{'}=\gamma_{1,j}{\frac{1}{2}g}_{k,j}^{'}+{\frac{1}{2}u}_{k,j}^{'}$  $x_{k}^{'}=\gamma_{1,j}g_{k,j}^{'}+u_{k,j}^{'}$ | (29) |
| --- | --- | --- |

| and | $\frac{1}{2}\left( y_{k,1}-y_{k,2} \right)=\beta_{1}\frac{1}{2}\left( x_{k,1}-x_{k,2} \right)+\frac{1}{2}\left( v_{k,1}-v_{k,2} \right)$  ${\frac{1}{2}y}_{k}^{'}=\beta_{1}\frac{1}{2}x_{k}^{'}+{\frac{1}{2}v}_{k,j}^{'}$  $y_{k}^{'}=\beta_{1}x_{k}^{'}+v_{k,j}^{'}$ | (30) |
| --- | --- | --- |

Therefore, the fixed effects estimator is equal to the first difference estimator with all of the variables included divided by 2. As this transformation is applied to every variable in the model it cancels out across the estimation and the estimated parameters will be the same as in the first difference model.

When there are more than two individuals in each family, the difference between these estimators depends on the error terms. If $v_{k,i,j}$ is assumed to be uncorrelated between individuals in a family, then the fixed effects estimator is more efficient. However, if it is assumed to be dependent on the level taken by other family members (i.e.$v_{k,1,j}= v_{k,2,j}+\epsilon_{k}$ where $\epsilon_{k}$ is a randomly distributed variable) then the first difference estimator is more efficient.^82^

**Supplementary Note 3: Analytic derivation of familial sources of bias as omitted variable bias**

**Dynastic effects (genetic nurture)**

Consider two parents, a father and mother, f and m who at a specific bivariate locus have one of two alleles of a given frequency:

|  | $g_{1f}\sim Bern\left( p \right)$  $g_{2f}\sim Bern\left( p \right)$  $g_{1m}\sim Bern\left( p \right)$  $g_{2m}\sim Bern\left( p \right)$ | (31) |
| --- | --- | --- |

Thus, at each locus, the parents can be one of three genotypes, homozygous 00, homozygous 11, or heterozygous 10. Each pair of parents have one offspring, which inherits one of the parents two alleles. The offspring inherit their alleles at random from their parents as indicated by the variable $t_{m},t_{f}\sim Bern\left( 0.5 \right)$:

|  | $g_{1i}=tg_{1f}+\left( 1-t \right)g_{1m}$  $g_{2i}=\left( 1-t \right)g_{2f}+tg_{2m}$  14 | (32) |
| --- | --- | --- |

The parents’ phenotypes are a function of their genotypes and a random environmental error term:

|  | $g_{1i}=t_{m}g_{1f}+\left( 1-t_{m} \right)g_{2f}$  $g_{2i}={t_{f}g}_{2f}+\left( 1-t_{f} \right)g_{2f}$ | (33) |
| --- | --- | --- |

Where $\beta$ is a constant, the paternal and maternal independent error terms are indicated by $e_{f}$ and $e_{m}$ $\sim N\left( \mu,\sigma^{2} \right)$, and constants are not shown. Similarly, the offspring phenotype function of the offspring genotype and an independent environmental error term. In addition, the offspring phenotype can also be affected by a dynastic, or genetic nurturing effect of the parental phenotype on the outcome indicated by $\gamma$:

|  | $p_{i}=\beta\left( g_{1i}+g_{2i} \right)+\gamma{\left( p_{f}+p_{m} \right)+e}_{i}$ | (34) |
| --- | --- | --- |

The maternal, paternal and offspring allele scores are $g_{m}=g_{1m}+g_{2m}$, $g_{f}=g_{1f}+g_{2f}$ and $g_{i}=g_{1i}+g_{2i}$ respectively, and $e_{i}$ $\sim N\left( \mu,\sigma^{2} \right)$.

The OLS estimator for $\beta$ is the equation for omitted variable bias:

|  | $\hat{\beta}_{ols}=\beta+\frac{\sum g_{i}\left( \gamma{\left( p_{f}+p_{m} \right)+e}_{i} \right)}{\sum\left( g_{i} \right)^{2}}$ | (35) |
| --- | --- | --- |

The error term $e_{i}$ is independent of $g_{i}$ so this can be simplified to:

|  | $\hat{\beta}_{ols}=\beta+\frac{\gamma\sum g_{i}\left( p_{f}+p_{m} \right)}{\sum\left( g_{i} \right)^{2}}$ | (36) |
| --- | --- | --- |

Substituting in for parental phenotypes:

|  | $\hat{\beta}_{ols}=\beta+\frac{\gamma\sum g_{i}\left( \beta g_{f}+e_{f}+\beta g_{m}+e_{m} \right)}{\sum\left( g_{i} \right)^{2}}$ | (37) |
| --- | --- | --- |

The error terms $e_{f}$ and $e_{m}$ are independent of $g_{i}$ so this can be simplified to:

|  | $\hat{\beta}_{ols}=\beta+\frac{\gamma\beta\sum g_{i}\left( g_{f}+g_{m} \right)}{\sum\left( g_{i} \right)^{2}}$ | (38) |
| --- | --- | --- |

The covariance between parental and offspring genotypes is equal to half the variance of the genotype, therefore:

|  | $\hat{\beta}_{ols}=\beta\left( 1+\gamma\right)$ | (39) |
| --- | --- | --- |

The bias in the OLS estimate will be:

|  | $bias\left( \hat{\beta}_{ols} \right)=\beta\gamma$ | (40) |
| --- | --- | --- |

This implies that the bias in the OLS estimate will be the product of the dynastic effect and the true causal effect of the genotype on the phenotype in the outcome.

**Cross trait assortative mating**

Less intuitive is that some patterns of assortative mating can induce SNP-outcome relationships that are due to bias, in that they do not arise due to a counterfactual allelic substitution occurring at the individual level. This is problematic for GWAS in general, but MR specifically if the SNP in question is being used to instrument the exposure under analysis. This has been examined through simulation in detail before, and here we examine how the bias arises from a theoretical perspective.^6^ Let $\rho$ be the correlation between the male exposure phenotype $x_{m}$ and the female outcome phenotype $y_{f}$ that arises due to assortment. If $x$ causes $y$, then any genetic influence on $x$ is also an influence on $y$, but for bias to arise we are interested in the possibility of there being an association between $y$ and the instruments for $x$ that is not through a biological causal relationship. To this end, suppose $x$ and $y$ are each heritable, and we have an instrument $g_{x}$ for $x$, let us define a genetic score for $y$, $s_{y}$, that is biologically independent of $g_{x}$. The system being analysed is:

|  | $x_{i}={\gamma_{i}g}_{x,i}+C_{i}+u_{i}$ $y_{i}={\beta_{i}x}_{i}+s_{y}+C_{i}+v_{i}$ | (41) |
| --- | --- | --- |

where C is a confounder, u and v are error terms, and $i\in\{m,f,o\}$ is used to denote the group of individuals in which the parameters are being estimated – males, females, offspring. The causal effect estimate is obtained as

|  | $\hat{\beta_{i}}=\frac{cov(g_{x,i},y_{i})}{cov(g_{x,i},x_{i})}$ | (42) |
| --- | --- | --- |

We know that due to Mendelian inheritance $cor(g_{m},g_{o})=cor(g_{f},g_{o})=0.5$. We can also infer that following assortment of male x and female y phenotypes, the expected covariance between their respective genetic factors will be

|  | $cov\left( g_{x,m},s_{y,f} \right)=\rho\cdot cov\left( g_{x,m},x_{m} \right)\cdot cov\left( s_{y,f},y_{f} \right)$ | (43) |
| --- | --- | --- |

The assortative mating induces a covariance between the genetic instrument for x and the genetic score for y that is biologically independent of x:

|  | $cov\left( g_{x,o},s_{y,o} \right)=cov\left( g_{x,m},s_{y,f} \right)\cdot cor\left( s_{y,f},s_{y,o} \right)\cdot cor\left( g_{x,m},g_{x,o} \right)$ $=\frac{1}{4}\cdot\rho\cdot cov\left( g_{x},x_{o} \right)\cdot cov\left( s_{y,o},y_{o} \right)$ | (44) |
| --- | --- | --- |

Given that there is a direct biological influence of $s_{y,o}$ on $y_{o}$, substituting back into the causal effect estimate we find that if $\rho\neq0$ then the Mendelian randomization estimate will be biased:

|  | $\hat{\beta}_{o}=\frac{cov\left( g_{x,o},y_{o} \right)+\frac{cov\left( g_{x,o},s_{y,o} \right)}{{cor\left( y_{o},s_{y,o} \right)}^{2}}}{cov\left( g_{x,o},x_{o} \right)}$ $=\frac{cov\left( g_{x,o},y_{o} \right)+\frac{\frac{1}{4}\cdot\rho\cdot cov\left( g_{x,o},x_{o} \right)\cdot var\left( y_{o} \right)var(s_{y,o})}{cov(s_{y,o},y_{o})}}{cov\left( g_{x,o},x_{o} \right)}$ | (45) |
| --- | --- | --- |

Simplifying to give a bias term:

|  | $=\frac{cov\left( g_{x,o},y_{o} \right)}{cov\left( g_{x,o},x_{o} \right)}+\frac{\rho\cdot cov\left( g_{x,o},x_{o} \right)\cdot var\left( y_{o} \right)var(s_{y,o})}{4\cdot cov(s_{y,o},y_{o})\cdot cov\left( g_{x,o},x_{o} \right)}$ | (46) |
| --- | --- | --- |

The left hand term here is the standard Mendelian randomization Wald estimate, the right hand expression in this formula is the bias term from assortative mating. This indicates that as the strength of assortment increases, the bias increases.

**Supplementary Figure 1**: Estimates of the effect of BMI on self-reported diabetes and high blood pressure and height and BMI on educational attainment using Mendelian randomization in samples of siblings using inverse variance weighted (IVW), weighted median, weighted modal and MR-Egger. This analysis uses a split sample approach, in which the SNP-exposure and SNP-outcome associations are estimated in separate samples and allow for a family fixed effect. The weighted median, weighted modal and MR-Egger estimators are less precise than IVW. We found little evidence of pleiotropy using MR-Egger. There was little evidence of heterogeneity between HUNT and UKBB for any of the estimates. The MR-Egger intercepts for all outcomes found little evidence of directional pleiotropy (p>0.05), however this may be due to lack of power. The total analysed sample size in UK Biobank and HUNT was 61,008. 95% confidence intervals reported.

**Supplementary Figure 2**: Replication of the results in 23andMe data. We re-estimated the SNP-phenotype associations in 223,368 individuals (111,684 families) from 23andMe. The analysis used 64 SNPs associated with BMI in Locke et al. (2015) and 347 SNP associated with height in Wood et al. (2014).^65,72^ 95% confidence intervals reported.

**Supplementary Figure 3**: A directed acyclic graph (DAG) illustrating the relationships and potential confounding mechanisms if the familial effects do not affect the outcome. In each case, there are no open paths from the SNP to the outcome, therefore familial effects that are mediated solely through the exposure are unlikely to cause bias.


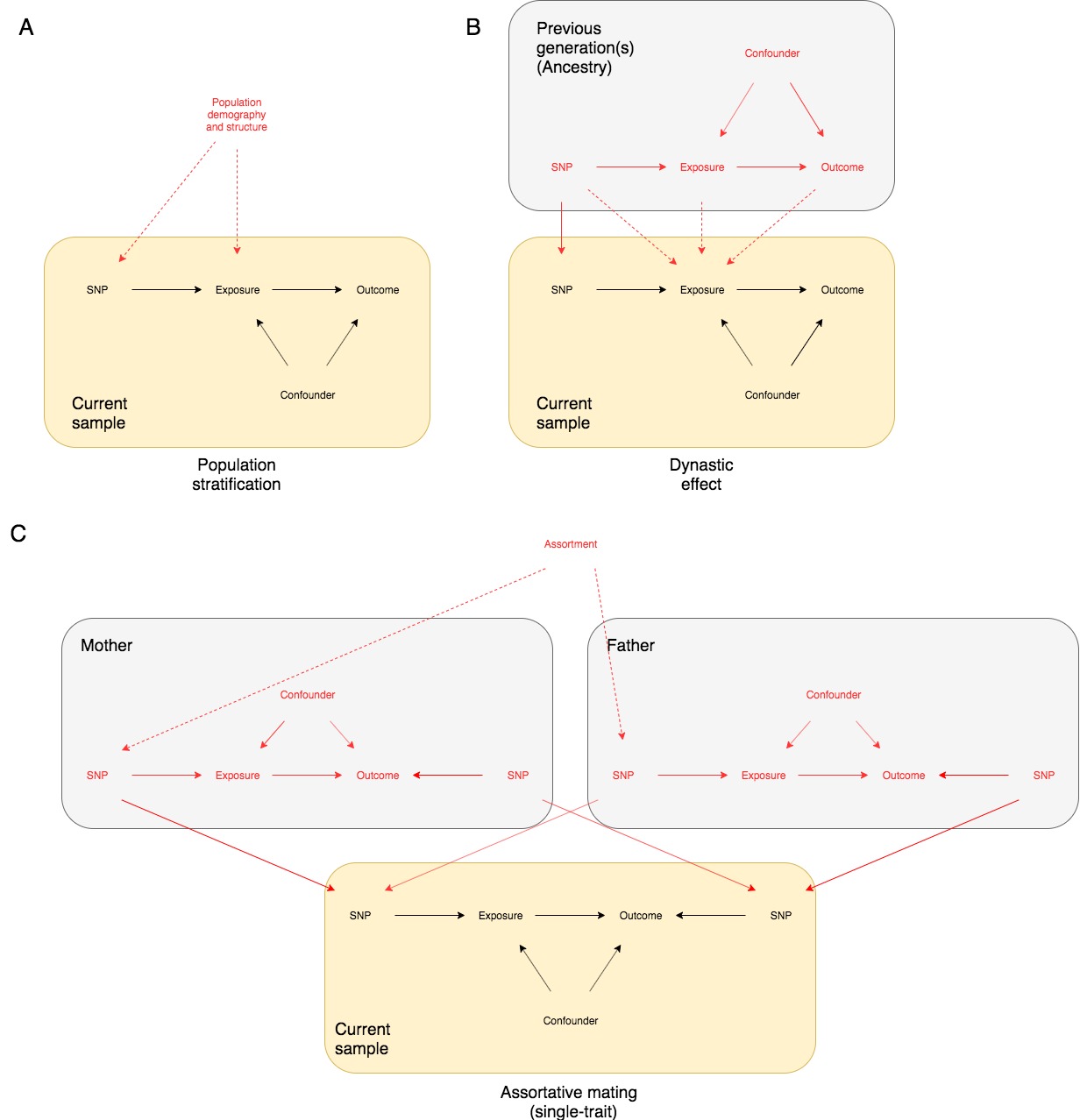


**Supplementary Figure 4**: STROBE Flow chart representing inclusion into the study sample for HUNT2.

Invited to participate in HUNT2 N = 93,898

Did not attend study clinic N=28,661

Attended study clinic and consented N=65,237

Did not meet genotyping QC thresholds

Non-European

Withdrew consent

Missing height or education

N=11,949

**Included sample**

No siblings

N=24,511

Siblings

N=28,777

Met criteria for inclusion N=53,288

**Supplementary Figure 5**: STROBE Flow chart representing inclusion and inclusion into the study sample for the UK Biobank.

**Included sample**

Invited to participate in UK Biobank N = 9,283,453

Attended study clinic and consented N=503,317

Met criteria for inclusion N= 367,575

Did not attend study clinic N=8,780,136

Did not meet genotyping QC thresholds

Non-white or non-British

Withdrew consent

Missing diabetes, high blood pressure, BMI height, or education,

N=135,742

Siblings

N=32,231

No siblings

N=335,344

**Supplementary Figure 6**: STROBE Flow chart representing inclusion and inclusion into the study sample for the 23andMe sample.

**Included sample**

Consented 23and Me research participants N = 5,957,233

Siblings N=626,609

Met criteria for inclusion N= 384,136

No siblings

N=5,330,624

Non-European

N=242,473

Eligible siblings

N=222,368

Incomplete data and restricting to two sibs per family

N=161,768

**Supplementary Table 1: The shrinkage of the within family SNP-phenotype associations. Estimated in UK Biobank and HUNT using seemingly unrelated regression.**

| Phenotype | Ratio | 95% Confidence interval |
| --- | --- | --- |
| Education | 0.432 | 0.356 to 0.508 |
| BMI | 0.821 | 0.760 to 0.881 |
| Height | 0.749 | 0.721 to 0.777 |
| High blood pressure | 0.721 | 0.627 to 0.814 |
| Diabetes | 0.888 | 0.789 to 0.987 |

**Supplementary Table 2.** Ratio of the standard errors with and without allowing for a familial effect for each of the empirical examples presented in Figure 3.

| Phenotype | Ratio of SEs of  MR-PRS family FE/MR-PRS siblings (increase in sample size required) | Ratio of SEs of  2SMR IVW siblings – split sample/MR-PRS siblings (increase in sample size required) |
| --- | --- | --- |
| BMI on diabetes | 1.71 (2.94) | 1.50 (2.24) |
| BMI on high blood pressure | 1.71 (2.93) | 1.49 (2.22) |
| Height on education | 1.57 (2.48) | 1.39 (1.94) |
| BMI on education | 1.45 (2.10) | 1.23 (1.50) |

**The Within Families Consortium**

*K.G. Jebsen Center for Genetic Epidemiology, Department of Public Health and Nursing, NTNU, Norwegian University of Science and Technology, Norway*

Ben Brumpton, Gunnhild Åberge Vie, Johan Håkon Bjørngaard, Kristian Hveem, Bjørn Olav Åsvold, Neil M Davies and Nicole M Warrington

*Medical Research Council Integrative Epidemiology Unit, University of Bristol, BS8 2BN, United Kingdom*

Ben Brumpton, Eleanor Sanderson, Fernando Pires Hartwig, Sean Harrison, Yoonsu Cho, Laura D Howe, Amanda Hughes, Alexandra Havdahl, Laurence Howe, Tim Morris, Frank Windmeijer, David M Evans, George Davey Smith, Gibran Hemani, Neil M Davies, and Claire Haworth

*Clinic of Thoracic and Occupational Medicine, St. Olavs Hospital, Trondheim University Hospital*

Ben Brumpton

*Population Health Sciences, Bristol Medical School, University of Bristol, Barley House, Oakfield Grove, Bristol, BS8 2BN, United Kingdom*

Ben Brumpton, Eleanor Sanderson, Sean Harrison, Yoonsu Cho, Laura D Howe, Amanda Hughes, Laurence Howe, Tim Morris, George Davey Smith, Gibran Hemani, Neil M Davies, Claire Haworth, and Laurie Hannigan

*23andMe, Inc., 223 N Mathilda Avenue, Sunnyvale, CA, USA 94086*

Karl Heilbron, Adam Auton

*Postgraduate Program in Epidemiology, Federal University of Pelotas, Pelotas, Brazil*

Fernando Pires Hartwig

*Netherlands Twin Register, Department of Biological Psychology, Vrije Universiteit Amsterdam, Amsterdam, The Netherlands*

Dorret I Boomsma, Michel G Nivard and Meike Bartels

*Nic Waals Institute, Lovisenberg Diaconal Hospital, Spångbergveien 25, 0853 Oslo, Norway*

Alexandra Havdahl and Laurie Hannigan

*Department of Mental Disorders, Norwegian Institute of Public Health, Sandakerveien 24C, 0473 Oslo, Norway*

Alexandra Havdahl

*The University of Melbourne, 207 Bouverie St, Carlton, Victoria, 3010, Australia*

John Hopper

*Virginia Institute for Psychiatric and Behavior Genetics, Virginia Commonwealth University, Richmond, Virginia*

Michael Neale

*Karolinska Institutet, Dept of Medical Epidemiology and Biostatistics, Stockholm, Sweden*

Nancy L Pedersen and Sara Hägg

*Department of Psychology, University of California Riverside, Riverside, CA, USA*

Chandra Renyolds

*Department of Psychology and Population Research Center, University of Texas at Austin*

Elliot M Tucker-Drob, Andrew Grotzinger and K Paige Harden

*Centre for Epidemiology and Biostatistics, Melbourne School of Population and Global Health, The University of Melbourne 207 Bouverie Street, Carlton, Victoria 3053, Australia*

Shuai Li

*Centre for Cancer Genetic Epidemiology, Department of Public Health and Primary Care, University of Cambridge, Strangeways Research Laboratory, Worts Causeway, Cambridge CB1 8RN, United Kingdom*

Shuai Li

*Department of Statistics, Nuffield College, University of Oxford, Oxford, OX1 3LB, United Kingdom*

Frank Windmeijer

*Nord University, Faculty of Nursing and Health Sciences, Levanger, Norway*

Johan Håkon Bjørngaard

*Center for public health genomics, Department of public health sciences, University of Virginia, Charlottesville, VA, USA*

Wei-Min Chen

*Department of Biostatistics and Center for Statistical Genetics, University of Michigan, Ann Arbor, USA*

Cristen Willer

*Department of Internal Medicine, University of Michigan, Ann Arbor, MI, USA*

Cristen Willer

*Department of Human Genetics, University of Michigan, Ann Arbor, USA*

Cristen Willer

*University of Queensland Diamantina Institute, University of Queensland, Brisbane, Queensland, Australia*

David M Evans

*Department of Public Health, University of Helsinki, Helsinki, Finland*

Jaakko Kaprio

*Institute for Molecular Medicine Finland (FIMM), University of Helsinki, Helsinki, Finland*

Jaakko Kaprio

*Department of Endocrinology, St Olavs Hospital, Trondheim University Hospital, Trondheim, Norway*

Bjørn Olav Åsvold

*School of Psychological Science, University of Bristol, Bristol, United Kingdom*

Claire Haworth

*University of Edinburgh, Centre for Genomic and Experimental Medicine, Institute of Genetics and Molecular Medicine, University of Edinburgh, Crewe Road, Edinburgh, UK EH4 2XU*

Archie Campbell, Andrew M McIntosh

*MRC Human Genetics Unit, Institute of Genetics and molecular Medicine, University of Edinburgh, University of Edinburgh*

Caroline Hayward, James F Wilson, Lucija Klaric

*Centre for Global Health Research, Usher Institute, University of Edinburgh, Teviot Place, Edinburgh, EH8 9AG, Scotland*

James F Wilson

*Institute of Genetics and Molecular Medicine, University of Edinburgh, Western General Hospital, Edinburgh, EH4 2XU, Scotland*

James F Wilson

*Institute for Molecular Medicine Finland (FIMM), P. O. Box 20 (Tukholmankatu 8) 00014 University of Helsinki*

Teemu Palviainen

*University of Minnesota, 75 East River Road, Minneapolis, MN 55455*

William Iacono

*The Danish Twin Registry, Department of Public Health, University of Southern Denmark, J.B. Winsloews Vej 9B, 5000 Odense C, Denmark*

Kaare Christensen and Marianne Nygaard

*Italian Twin Register, Center for Behavioural Sciences and Mental Health, Istituto Superiore di Sanità Viale Regina Elena 299, I-00161 Rome, Italy*

Maria Antonietta Stazi and Sonia Brescianini

*Faculty of Psychology and IMIB-Arrixaca. University of Murcia. Campus de Espinardo. 30100 Murcia (Spain)*

Juan R Ordoñana

*The University of Melbourne 207 Bouverie St, Carlton, Victoria, 3010, Australia*

Maria Antontietta Stazi

*GRIP, École de psychologie, Université Laval Pavillon Félix-Antoine-Savard, 2325 Allée des Bibliothèques, Québec, QC G1V 0A6, Canada*

Michel Boivin

*Dept of Medical Epidemiology and Biostatistics , Karolinska Institutet, Stockholm, Sweden*

Nancy L Pedersen

*College of Medicine and Health, University of Exeter, RILD Building, Barrack Road, Exeter, EX2 5DW*

Rachel M Freathy

*Institute of Biomedical and Clinical Science, University of Exeter, Exeter, UK*

Robin N Beaumont

*QIMR Berghofer Medical Research Institute, University of Queensland, Brisbane, Queensland, Australia*

Sarah E Medland and Nick Martin

*University of Queensland Diamantina Institute, University of Queensland, Brisbane, Queensland, Australia*

Nicole M Warrington

*Department of Epidemiology, School of Public Health, University of Michigan, 1415 Washington Heights, Ann Arbor, MI 48109, USA*

Patricia A Peyser and Lawrence F Bielak

*ATGU, Massachusetts General Hospital*

Patrick Turley

*The Stanley Center, Broad Institute*

Patrick Turley

*Medical Research Council Population Health Research Unit, Nuffield Department of Population Health, University of Oxford*

Iona Y Millwood

*Clinical Trials Service Unit and Epidemiological Studies Unit, Nuffield Department of Population Health, University of Oxford*

Iona Y Millwood

*Centre for Fertility and Health, Norwegian Institute of Public Health, Oslo, Norway*

Fartein Ask Torvik

*Department of Clinical, Educational and Health Psychology, University College London*

Jean-Baptiste Pingault

*Social, Genetic, and Developmental Psychiatry Centre, King's College London*

Jean-Baptiste Pingault

*Glenn Biggs Institute for Alzheimer and Neurodegenerative Diseases, University of Texas Health Sciences Center, San Antonio, TX*

Sudha Seshadri

*Framingham Heart Study, Framingham, MA*

Sudha Seshadri

*Boston University School of Medicine*

Sudha Seshadri

*Leverhulme Centre for Demographic Science, University of Oxford*

Melinda C Mills

*Nuffield College, University of Oxford*

Melinda C Mills

*Nuffield Department of Population Health, University of Oxford, Oxford OX3 7LF, UK*

Robin G. Walters

*MRC Population Health Research Unit, University of Oxford, Oxford OX3 7LF, UK*

Robin G. Walters

*Lothian Birth Cohorts, Department of Psychology, University of Edinburgh, 7 George Square, Edinburgh EH8 9JZ, UK*

W. David Hill

*Amsterdam Public Health Research Institute, Amsterdam UMC, The Netherlands*

Meike Bartels

School of Business and Economics, Department of Economics, Vrije Universiteit Amsterdam, De Boelelaan 1105, 1081 HV, Amsterdam, Netherlands

Richard Karlsson Linnér, Philipp D. Koellinger and Aysu Okbay

Institute for Behavioral Genetics, University of Colorado Boulder

Matthew C Keller, Jared V Balbona and Yongkang Kim

*Institute of Health and Society, University of Oslo, P.O. box 1130, Blindern, 0318 Oslo, Norway*

Øyivnd Næss and Sudheer Giddaluru

*The 23andMe Research Team*

Michelle Agee, Stella Aslibekyan, Robert K Bell, Katarzyna Bryc, Sarah K Clark, Sarah L Elson, Kipper Fletez-Brant, Pierre Fontanillas, Nicholas A Furlotte, Pooja M Gandhi, Barry Hicks, David A Hinds, Karen E Huber, Ethan M Jewett, Yunxuan Jiang, Aaron Kleinman, Keng-Han Lin, Nadia K Litterman, Marie K Luff, Matthew H McIntyre, Kimberly F McManus, Joanna L Mountain, Sahar V Mozaffari, Priyanka Nandakumar, Elizabeth S Noblin, Carrie AM Northover, Jared O'Connell, Steven J Pitts, G. David Poznik, J Fah Sathirapongsasuti, Janie F Shelton, Suyash Shringarpure, Chao Tian, Joyce Y Tung, Robert J Tunney, Vladimir Vacic, and Xin Wang.
